# Lobe placentaire accessoire: penser aux vaissaux praevia

**DOI:** 10.11604/pamj.2017.26.4.10624

**Published:** 2017-01-04

**Authors:** Mehdi Kehila, Manel Seboui

**Affiliations:** 1 Service C du Centre de Maternité et de Néonatologie de Tunis, Faculté de Médecine de Tunis, Université Tunis El Manar, Tunisie

**Keywords:** Métrorragies, échographie transvaginale, lobe accessoire, vaisseau prævia, Metrorrhagia, transvaginal ultrasound, accessory lobe, vasa prævia

## Image en médecine

Il s’agit d’une patiente âgée de 36 ans, deuxième geste, deuxième pare hospitalisée à deux reprises pour des métrorragies au troisième trimestre de la grossesse. A chaque hospitalisation, une échographie a été pratiquée par voie sus-pubienne et a conclu à un placenta antérieur normalement inséré sans images de décollement. A l’occasion de la troisième hospitalisation, à 36 semaines d’aménorrhée, une échographie transvaginale a été réalisée. Celle-ci a révélé une structure d’échogénicité similaire à celle du placenta, située sur la face postérieure de l’utérus, à 24mm de l’orifice interne du col, évocatrice d’un lobe placentaire accessoire. L’application du Doppler couleur a montré un vasa prævia connecté à ce lobe. Une césarienne a alors été programmée à 37SA donnant naissance à un bébé de sexe masculin, pesant 3800g en bon état de santé. L’étude macroscopique du placenta a montré le lobe placentaire accessoire séparé du principal disque placentaire par des membranes et relié à celui-ci par de larges vaisseaux.

**Figure 1:**
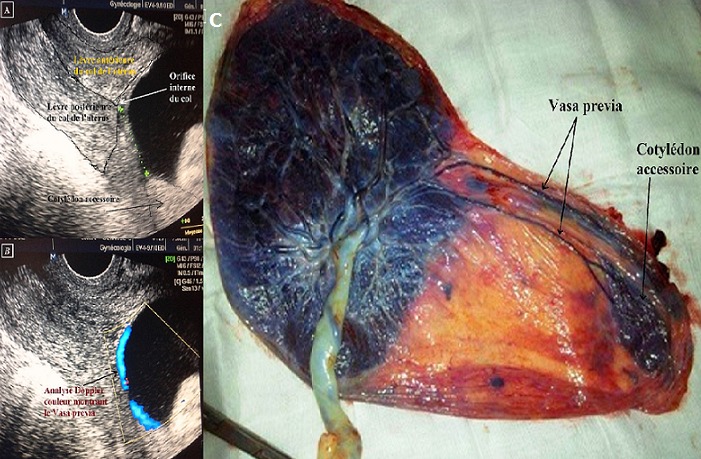
A) échographie endovaginale montrant un lobe accessoire postérieur à 24 mm de l’orifice interne du col; B) analyse Doppler montrant un vaisseau preavia; C) aspect macroscopique montrant le lobe accessoire relié au placenta par de larges vaisseaux

